# NK Cells and MSCs: Possible Implications for MSC Therapy in Renal Transplantation

**DOI:** 10.4172/2157-7633.1000166

**Published:** 2014-02-07

**Authors:** Marlies EJ Reinders, Martin J Hoogduijn

**Affiliations:** 1 Department of Nephrology, Leiden University Medical Center, Leiden, The Netherlands; 2 Transplantation and Nephrology, Internal Medicine, Erasmus MC, Rotterdam, The Netherlands

**Keywords:** MSC, NK cells, Transplantation

## Introduction

Kidney transplantation has improved survival and quality of life for patients with end-stage renal disease. The first year results of kidney transplantation have significantly improved over the years and patient and renal transplant survival are now >90%. However, long-term graft survival has remained virtually similar over the last decades [1]. Indeed, in the United States and Europe, thousands of kidney transplants fail each year, (and kidney-allograft failure is a major cause of end-stage renal disease,) leading to increased morbidity, mortality, and costs. It has become evident that ongoing alloreactivity, both cellular and humoral, plays a critical role in the loss of kidney allografts. In addition to the usual suspects, T cells and B cells, it has been acknowledged recently that Natural Killer (NK) cells play an important role in both innate and adaptive immune responses against the allograft and modulation of their function might improve transplant outcome [2]. NK cells are however poorly controllable with conventional immunosuppressive drugs and therefore there is a need for alternative methods to target these cells. In the last decades, basic and clinical interest in Mesenchymal Stromal Cells (MSCs) has risen dramatically due to their immune suppressive and reparative properties [3]. MSCs are characterized by their ability 1) to adhere to plastic culture flasks; 2) the positive expression of CD105, CD73, CD90 membrane antigens, and the lack of expression of others (e.g CD45 and CD34) and 3) the ability for differentiation in osteogenic, chondrogenic and adipogenic lineages [4]. MSCs are indeed potent immune suppressive cells and are able to constrain severe immunologic responses both in experimental models and in clinical conditions such as transplant rejection [3,5]. MSCs have also been shown to modulate NK cell responses [6] which might be of importance for the fate of the allograft. In this report the role of NK cells in renal transplantation, the interaction between MSCs and NK cells and its possible implications for renal transplantation is being commented.

## The Role of NK Cells in Transplantation

Natural killer cells are a subset of lymphocytes which traditionally have been seen as a central compartment of the innate immune system. Recent papers show that NK cells can discriminate between cells of self and foreign origins and that they also may play key roles in adaptive immune responses [2]. Although in earlier NK cell depletion studies it was thought that NK cells may not contribute to early acute rejection of solid organ allografts, recent studies demonstrate that NK cells play a pivotal role in acute and chronic rejection. This controversy was probably due to the initial depletion studies with selected knocked out genes that lead to simplistic and erroneous conclusions [2]. NK cells can kill allogeneic target cells directly or via Antibody-Dependent Cellular Cytotoxicity (ADCC) in the absence of antigen priming [7,8]. In addition, the innate component of NK cell immune reactivity may contribute to anti-allograft immune responses inflicted by ischemia-reperfusion induced organ injury. NK cells can release various chemokines and immune modulatory cytokines necessary for graft infiltration of immune cells including IFN-γ, of importance for enhancing Th1 cell alloreactivity. On the other hand, NK cells may also play a central role in regulation of alloimmunity and tolerance to allogeneic organ transplants by the production of Interleukin (IL)-10 and subsequent type 2 cytokine immune deviation as well as stimulation of regulatory T cells [2]. In addition, NK cells may directly eliminate donor APCs. Depending upon the types of NK cell receptors engaged and the microenvironment, early NK cell activation can thus promote either acute rejection or tolerance.

It is therefore of importance to better characterize receptors, ligands and cytokines involved in NK functions and to gain more insight into the mechanisms by which NK cell activation can be modulated in order to find tools to use NK cells to improve the fate of the allograft. MSCs may provide such a tool.

## Immunomodulatory Properties of MSCs: Complex Interplay between MSCs and NK Cells

**Figure 1:**
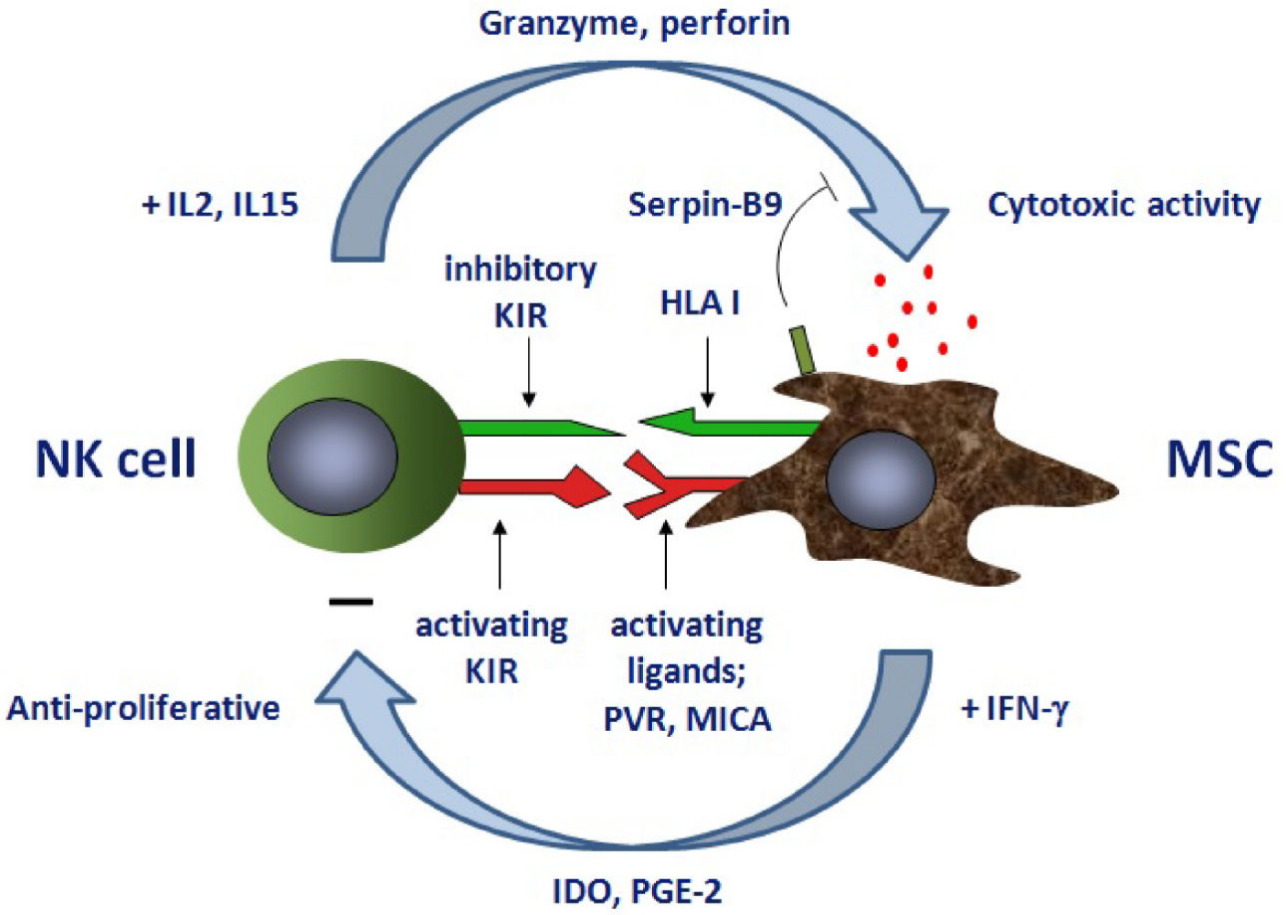
Schedule of the interaction between MSC and NKcells. MSC inhibit NK cell proliferation upon IFN-γ activation via amongst others IDO activity and PGE-2 secretion. IL2/IL15 activated NK cells upregulate granzyme and perforin expression and can lyse allogenic as well as autologous MSC. Lysis depends on the balanced expression of activating and inhibitory NK cell receptor ligands on MSC. Serpin-B9on MSC can inhibit the granzyme activity and there by protects MSC from NK cell lysis. **Abbreviations:** IDO: Indolamine2,3-Dioxygenase; KIR: killer Cell Immunoglobulin-like Receptor; MICA: MHC class I Polypeptide-related Sequence A; PGE-2: Prostaglandin-E2; PVR: Poliovirus Receptor; Serpin-B9: Serineprotease b9.

MSCs and NK cells interact in a complex manner (Figure 1). Culture expanded MSCs are recognized and killed by both allogeneic and autologous IL-2 or IL-15 activated NK cells [6,9,10]. The low MHC class I expression on MSCs and the expression of activating NK cell receptor ligands, such as Poliovirus Receptor (PVR) and MHC class I polypeptide-related sequence A (MICA), makes them a natural target for activated NK cell killing [6,10,11]. This can be partially overcome by incubating MSC with IFN-γ, which up-regulates MHC class I expression [6,11]. MSCs have been shown to possess several mechanisms to counteract their susceptibility to NK mediated killing, including Serine Protease Inhibitor 9 (SERPINB9), which is a major defense against granzyme B-mediated lysis [12]. Recently it was shown that Toll like Receptors (TLR) may modulate the interaction between MSC and NK cells. MSC express a number of different TLRs (Delarosa Frontiers 2012) and depending on which TLR is activated, the susceptibility of MSC for NK cell lysis or the suppressive effects of MSC on NK cell function are modulated. TLR3 activation protects MSC from NK cell killing and leads to enhanced immunosuppressive function of MSC against NK cells [13]. In contrast, TLR2 stimulation reduces the immunosuppressive property of MSC [14]. TLR3/4 activation of MSC stimulates the recruitment of inflammatory cells and in combination with IFN-γ may increase immune responses induced by MSCs [15]. TLR pre-stimulation may thus represent a possible means to induce the suppression of NK cell activity by MSC and reduce the lysis of MSCs by NK cells. Optimization of MSC therapy may thus require reduction of NK cell activity. On the other hand, removal of infused MSCs may be of major importance for safety of MSC therapy. It is however not clear yet whether longevity of MSCs after infusion is of importance for the efficacy of MSC therapy.

Whereas they can be targets for NK cells, MSCs can also strongly alter NK cell phenotype and suppress cytokine secretion and cytotoxicity against HLA class I expressing targets [6,10,16]. It is demonstrated that IDO and PGE2 are key mediators of MSC-induced inhibition of NK cells [16]. Other factors such as IL-10, TGF-β and HGF may play additional roles [17]. Interestingly, the immunosuppressive effect of MSC on recipient anti-donor reactivity was examined before and after human kidney transplantation in vitro. MSC inhibited the proliferation of not only CD4+ and CD8+ T-cell lymphocytes in pre and post-transplant donor directed MLR, but also of NK cells [18]. The inhibition of NK cell proliferation was shown to be merely dependent of soluble factors. The ant proliferative effect of MSC is induced under inflammatory conditions, such as in microenvironments rich in IFN-γ.

The interaction between MSCs and NK cells is thus interplay between the inhibition of NK cell function by MSCs and the cytotoxic attack of MSCs by NK cells. One of the mechanisms of NK-MSC interaction is mediated by activating ligands expressed by MSCs and activating receptors on NK cells [19]. Natural Killer Group 2d (NKG2D) is critically involved in the recognition of stress-inducible MHC class I chain related protein (MIC)-A, MICB and the UL16-Binding Proteins (ULBP) 1-4 family expressed on NK/MSC interaction [20]. Of importance, activated NK cells can trigger organ rejection through NKG2D and affect the maturation of APCs and ultimately the T-cell allogeneic response [21]. The further elucidation of mechanisms of MSC-NK crosstalk remains an important research goal for future studies.

## MSCs in Experimental Models of Organ Transplantation: What is the Link with NK Cells?

An attractive potential indication for MSC therapy in view of its potent effects on immune cells, is solid organ transplantation [22,23]. MSCs may potentially not only allow for reduced doses of immune suppressive drugs, but may also promote immune tolerance [24-27]. Different models have found prolongation of allograft survival after MSC treatment [28-30] and an inhibition of the rejection process [31,32]. The study by Franquesa et al. [33] investigated the effect of MSCs on the fibrosis reaction that occurs long after transplantation. MSCs attenuated the progression of Interstitial Fibrosis/Tubular Atrophy (IF/TA) when this process was already in progress. Besides a reduction in IF/TA, MSC treated animals demonstrated less macrophage infiltration in the parenchyma and lowered expression of inflammatory cytokines [33]. These results suggest that the beneficial effect of MSC on the outcome of organ transplantation is attributable to the reduction of immune reactivity by MSCs, rather than by promoting tissue regeneration as previously thought. However, since fibrosis and inflammation are interconnected it remains difficult to dissect the different mechanisms involved. To study the *in vivo* immunosuppressive effect in a humanized transplant model, we recently used a humanized skin transplantation model. In this model, alloreactivity was marked by pronounced CD45+ T-cell infiltrates consisting of CD4+ and CD8+ T cells and increased IFNγ expression in the skin grafts, which were all significantly inhibited by both BM-MSC and ASC treatment. Our findings show that BM-MSC and ASC are immunosuppressive in vitro and suppress alloreactivity in a preclinical humanized transplantation model [48].

Although numerous recent reports have focused on both the role of MSCs and NK cells in experimental models of solid organ transplantation, the role of NK cells in MCS mediated responses and vice versa in these models has been largely overlooked. In vivo, in a recent study by Almeida et al. [34] the interaction between NK cells and MSCs was investigated in an inflammatory model of biomaterial incorporation. Enhanced MSC recruitment was found to be mediated via NK cells by incorporation of inflammatory signals in the biomaterials. In addition, a recent study demonstrated that MSC decrease the number of infiltrating NK cells in ischemic hind limbs [35]. These results are interesting also for the transplant setting, and indicate that MSC can target NK cells in ischemic transplanted organs. Of importance murine and human MSCs exhibit different properties and express different cytokines [36], thereby challenging the validity of murine in vivo models for the preclinical evaluation of human MSC.

## MSCs Clinical Studies

In the human transplant setting different studies have focused on the role of MSCs in the induction phase and in allograft rejection. MSC infusion was safe and clinical feasible [37-39], although timing of the infusion seemed of major importance. Administration of MSC in the early phase after transplantation negatively affected kidney graft function, which was not observed when MSCs were administered before transplantation [38]. In a recent clinical pilot study allogeneic MSCs were administered in 6 renal recipients for the first time. Donor derived MSCs combined with low dose tacrolimus was safe and prevented acute rejection after renal transplantation [40]. In a phase 1 clinical study, safety and feasibility of autologous MSC therapy in patients receiving an HLA-DR mismatched kidney was studied in patients with subclinical rejection or an increase in IFTA in their renal biopsy at 24 weeks after renal transplantation (compared to the renal biopsy at 4 weeks). In total 6 patients received MSC treatment [41] and initial results suggested systemic immune suppression after MSC therapy. All patients that received MSCs demonstrated a profound reduction in proliferation of patient Peripheral Blood Mononuclear Cells (PBMC) 12 weeks after MSC infusion upon stimulation with donor specific PBMCs, while the response to third party PBMCs was more variable. In addition, three patients developed opportunistic viral infections (2 CMV, 1 BK nephropathy) and two patients with allograft rejection demonstrated a resolution of infiltrates after MSC treatment. In the treated patients, amounts of different immune cells, including NK cells, were measured before and after MSC treatment. Total numbers and subsets of NK cells, T cells, B cells and monocytes did not demonstrate a consistent change after MSC treatment. Since only 6 patients were treated, larger studies are needed to draw conclusions.

Most clinical studies so far have used autologous MSC. While MSC were long considered to be immunoprivileged, there is accumulating data that they can evoke immune responses [42-49]. Nevertheless, allogeneic MSCs offer the advantage of availability for clinical use without the delay required for expansion. This is of major importance in the case of indications where immediate treatment is needed, for example in allograft rejection of the transplanted organ. HLA class I on allo MSCs may trigger adaptive immune responses that could possibly elicit an anti- donor immune response, which may increase the incidence of rejection/graft loss and impact the allograft survival on the long term. NK cells however, identify their target cells in a different way. NK cell-mediated lysis of MSC is inversely correlated with expression levels of HLA class I on MSCs. Thus both autologous and allogeneic MSC with low HLA class I expression are potential targets for NK cells. In addition, there is possibly a difference in the susceptibility of autologous and allogeneic MSCS for lysis by NK cells. Killer Cell Immunoglobulin like Receptors (KIR) that does not recognize their KIR epitopes on allogeneic cells may lyse these cells [45]. It is not unlikely that NK cells interact with MSC in a similar manner. Therefore, allogeneic MSC may be more susceptible for NK cell lysis than autologous MSC.

As stated above, the conventional immunosuppressive drug used in organ transplantation target T cells and more recently B cell targeting drugs win ground. These drugs are however not capable of inhibiting NK cell activity. It was demonstrated that tacrolimus, rapamycin or sotrastaurin did not inhibit the lysis of autologous or allogeneic MSC by activated NK cells [46], whereas these drugs are effective in suppressing T cell reactivity. This indicates that drugs used to suppress allo-reactivity in transplant patients may be effective in inhibiting T cell reactivity against allogeneic MSC, but are not able to suppress NK cell mediated lysis of allogeneic and autologous MSC. Interestingly, a recent paper demonstrated that azathioprine can inhibit lysis of MSC by activated NK cells [47], suggesting that azathioprine might have a beneficial effect on the survival of MSC upon exposure to NK cells and improve the effect of MSC therapy.

## Conclusion

Taken together, so far, it has been challenging to describe the precise roles for NK cells in reactivity to allografts and the cross link between MSCs and NK cells makes this challenge even more complex. The microenvironment seems to be of major importance for both MSC and NK cell function and for their interaction. It can be postulated that a microenvironment rich in IFN-γ would protect MSC from being attacked and destroyed by NK cells. Such microenvironment may be found in organ transplants that face rejection. Since MSCs can also strongly alter NK cell phenotype and function, treatment with MSCs might be helpful in creating a pro tolerogenic microenvironment for NK cells which might improve allograft survival. In addition, both NK cells and MSCs may use the same pathways to regulate immune responses in transplant recipients. It will be an ongoing challenge to elucidate the exact mechanisms and cross-talk between MSCs and NK cells and their implications for organ survival.
